# Observations Respecting the Pulse

**Published:** 1819-06

**Authors:** Philo Physiologiæ


*For the London Medical and Physical Journal.*

*Observations respecting the Pulse;* by PHILO PHYSIOLOGIÆ.

IT is equally true with regard to researches in science as with the more light and amusing occupations of human life, that the pleasure we derive from favourite pursuits animates in return the imagination, and lends to the subjects of them those vivid tints, the effect of which is like the beams of the moon on the prominences of the Alps, that add to the apparent grandeur of the objects over which they are emanated, whilst the surrounding scene is veiled in mists, or concealed from accurate observance by the shades of night; and, on contemplating them thus, we behold them with such a degree of admiration as they do not elicit from those who view the whole of nature as illustrated by the light of day. That such may be the case with regard to myself on subjects relative to the physiology of organized beings, I am well disposed to acknowledge; and I mention this as an apology for the undue importance I may, perhaps, attach to the questions respecting the *Pulse*, which were a short time since proposed by your correspondent, Mr. Littleton. I have hitherto neglected to offer such replies to those questions as my literary acquirements will enable me to adduce, from a hope that some person better qualified to treat on this subject might think it not unworthy of his pen; and it is in default of this, that I shall detail what will, probably, not be novel to Mr. Littleton.

The only author, besides Dr. Blackall, with whom I am acquainted, who describes the *redoubling pulse*, is Theophilus Bordeu. After stating the character of the diseases of which it is usually a sign, that physician observes—

"Cette reduplication ne paroit étre que le fond d'une seule pulsation pártagée en deux temps ou en deux pulsations; elle est sujette à laisser de temps en temps des intervalles; ces intervalles sont plus ou moins longs, ou plus ou moins fréquens, selon la nature ou le degré de la maladie.

"Cette dilatation, qui se fait en deux temps ou par un double effort, paroit assez comparable à l'effet d'un piston qui pousseroit une liqueur dans un cylindre elastique, de manière que le second jet de la liqueur n'attendit pas que le premier se fût répandre dans le vaìsseau." And he again says,

"C'est une dilatation qui devroit se faire naturellement en un temps, qui cependant se fait en deux temps ou par deux efforts sensìbles, et qui succède à une contraction naturelle de l'artère."*

This, as Mr. Littleton suggests, is the same with the *pulsus dicrotus* of Galen and Solano de Lucque, and which, in his translation of the latter author, Nihell terms the *rebounding* pulse: that it is the same, is evident from the description of its character.

Having replied to the questions of Mr. Littleton, I may, perhaps, be allowed to adduce a few additional observations. The Chinese physicians describe this sort of pulse in a very forcible manner, *as that which moves like a serpent in doubling itself.*† All the authors whom I have mentioned state, that it indicates the occurrence of hæmorrhage, or of some critical evacuation, especially from the lungs. Solano de Lucque calculates the interval that will elapse before the former will ensue, from the greater or less frequency of this redoubling of the pulse; and states, that when it occurs at every systole of the heart, the evacuation will take place within twenty-four hours. Galen considered it as indicative of plethora, excitation of the system, and undue firmness of the small arteries.

I would by no means advocate the propriety of the subtle distinctions made by Galen, Herophilus, and the Chinese physicians, of the phenomena of the pulse; but still I think that they are too much confounded by the generality of modern physicians. The chief divisions of Galen: the strong, weak, frequent, slow, full, small, hard, and soft, pulses; comprise all the varieties usually spoken of by modern authors. Every experienced observer must notice various other characters, and is probably prevented describ-

* *Récherches sur le Pouls*, ch. v.

† See *les Sécrets de la Médecine des Chinois, consistant en la parfaite Connoissance du Pouls.* Grenoble, 1671.

ing them only from deference to custom. In the Chinese work, to which I have referred, there are several distinctions which, I believe, would furnish useful and decisive indications for the diagnosis of many diseases. I shall terminate this paper with a detail of the whole. There are four chief distinctions: the superficial, profound, quick, and slow. The varieties are: the full, empty, solid, large, fine, resounding, relaxed, rounded, uneven, long, short, raised, depressed, contracted or confined, weak, lively, hidden, precipitated, filled, suffocated, soft, hard, re-enforced, bound, intermittent, deceitful.

They use also the following metaphorical descriptions, which, although perhaps more curious than useful, evince much acuteness of observation: The pulse that moves like a serpent, in doubling its body; with inquietude, like a jay; like a frog out of the water; like a fowl, to one and the other side alternately; like a partridge, which runs a little, stops, and then hides itself; like a sparrow, hopping along a little way, and then stopping a little; skipping, like a goat; like a fish in the water; as if ants were creeping under the fingers. When we consider the minute directions the Chinese physicians give for the mode in which the pulse should be felt; the delicacy of touch they state to be requisite; and the particular pressure that should be made with each finger respectively and distinctly; we cannot be surprised that, with these notions, and their want of physiological knowledge, and consequently the greater necessity they have for an accurate observance of phenomena, they have been led to form the above subtle distinctions and metaphorical descriptions of phenomena of diseases, from which much and various useful information may doubtless be derived.

*London; April* 23, 1819.

